# RETRACTED: Anohova et al. The *Dosidicus gigas* Collagen for Scaffold Preparation and Cell Cultivation: Mechanical and Physicochemical Properties, Morphology, Composition and Cell Viability. *Polymers* 2023, *15*, 1220

**DOI:** 10.3390/polym16111512

**Published:** 2024-05-27

**Authors:** Veronika Anohova, Lyudmila Asyakina, Olga Babich, Olga Dikaya, Aleksandr Goikhman, Ksenia Maksimova, Margarita Grechkina, Maxim Korobenkov, Diana Burkova, Aleksandr Barannikov, Anton Narikovich, Evgeny Chupakhin, Anatoly Snigirev, Sergey Antipov

**Affiliations:** 1 Immanuel Kant Baltic Federal University, Nevskogo 14, Kaliningrad 236006, Russia; anohovaveronika@yandex.ru (V.A.); alk_kem@mail.ru (L.A.); olich.43@mail.ru (O.B.); ODikaya@kantiana.ru (O.D.); AGoikhman@kantiana.ru (A.G.); KMaksimova@kantiana.ru (K.M.); korobenkovmv@gmail.com (M.K.); abarannikov95@gamil.com (A.B.); narikovich@gmail.com (A.N.); chupakhinevgen@gmail.com (E.C.); anatoly.snigirev@gmail.com (A.S.); 2 Voronezh State University, 1, University Square, Voronezh 394063, Russia; grechkina@phys.vsu.ru (M.G.); diana.burkova488@gmail.com (D.B.); 3 Saint Petersburg State University, Saint Petersburg 199034, Russia

The *Polymers* Editorial Office retracts the article, “The *Dosidicus gigas* Collagen for Scaffold Preparation and Cell Cultivation: Mechanical and Physicochemical Properties, Morphology, Composition and Cell Viability” [1], cited above. 

Following publication, concerns were brought to the attention of the publisher regarding an overlap with previously published work from different authorship groups. 

Adhering to our complaints procedure, an investigation was conducted by the Editorial Office that confirmed an overlap of raw data originating from previously published studies [2,3] from different authorship groups, without appropriate citation or permission. As a result, the Editorial Office and Editorial Board have decided to retract this article [1] as per MDPI’s retraction policy (https://www.mdpi.com/ethics#_bookmark30). 

This retraction was approved by the Editor-in-Chief of the journal *Polymers*.

The authors agreed to this retraction.
